# Herpes zoster in Germany: Quantifying the burden of disease

**DOI:** 10.1186/1471-2334-11-173

**Published:** 2011-06-16

**Authors:** Bernhard Ultsch, Anette Siedler, Thorsten Rieck, Thomas Reinhold, Gérard Krause, Ole Wichmann

**Affiliations:** 1 Immunization Unit, Robert Koch Institute, 13086 Berlin, Germany; 2 Charité - University Medical Center, 10117 Berlin, Germany; 3 Surveillance Unit, Robert Koch Institute, 13086 Berlin, Germany; 4 Institute for Social Medicine, Epidemiology and Health Economics, Charité - University Medical Center, 10117 Berlin, Germany; 5 Department for Infectious Disease Epidemiology, Robert Koch Institute, 13086 Berlin, Germany

## Abstract

**Background:**

Herpes zoster (HZ) is caused by a reactivation of the varicella-zoster-virus (VZV) and mainly affects individuals aged ≥ 50 years. Vaccines have been licensed or are under development that can protect against HZ and its main complication postherpetic neuralgia (PHN). In Germany, the burden of disease caused by HZ is not well known. To support the decision making process related to a potential vaccination recommendation, we estimated annual HZ disease burden in people aged ≥ 50 years in Germany by utilizing various data sources.

**Methods:**

We assessed for 2007 and 2008 HZ-outpatient incidence (number of cases per 1,000 person-years, PY) by utilizing the Association of Statutory Health Insurance Physicians (ASHIP) database, which contains nationwide routine outpatient data. For the same time period annual number of HZ-inpatients and HZ-associated deaths were identified by using the Federal Health Monitoring System (FHM). PHN-incidence and loss of quality-adjusted life years (QALYs) caused by HZ were calculated by multiplying number of identified HZ-patients with upper and lower limit estimates for proportion of HZ-cases developing PHN and HZ-related QALY, respectively.

**Results:**

For the study period we identified an annual average of 306,511 HZ-outpatients aged 50+, resulting in a HZ-incidence of 9.6/1,000 PY. A total 14,249 HZ-associated inpatients and 66 deaths were reported in both years on average. HZ-incidence increased by age from 6.21 in people 50-54 years to 13.19 per 1,000 PY in people aged ≥ 90 years. Females were significantly more frequently affected than males in terms of outpatient HZ-incidence (11.12 vs. 7.8 per 1,000 PY), inpatient HZ-incidence (0.51 vs. 0.38 per 1,000 PY) and mortality (0.29 vs. 0.10 per 100,000 PY). PHN-incidence was estimated to range between 0.43 and 1.33 per 1,000 PY. Based on these figures, there were between 3,065 to 24,094 QALYs lost due to HZ in persons aged ≥ 50 years in Germany per annum.

**Conclusion:**

Our study provides important baseline estimates for HZ-related disease burden in Germany. HZ poses a considerable burden on the health care system in Germany both in terms of outpatient and inpatient services. Follow-up assessments of HZ disease burden are needed to monitor the impact of VZV-vaccinations in Germany.

## Background

Infection with the varicella-zoster-virus (VZV) usually occurs in childhood and causes chickenpox [1]. The virus persists lifelong in the dorsal roots of the cranial and spinal ganglia of humans. Especially in the elderly, the virus can reactivate as herpes zoster (HZ) due to decreasing VZV-specific T-cell-immunity [2,3]. Besides a 20-30% risk of developing HZ over lifetime, the risk of HZ increases distinctly from the fifth decade in life [1,4-6]. The main complication of HZ is postherpetic neuralgia (PHN), a long-lasting and occasionally recurring pain [3,6,7]. Both HZ and PHN cause limitation of the quality of life of affected individuals [8-11]. Thitherto the current treatment options of HZ and the prevention of PHN are not always optimal [12-14].

One vaccine for the protection against the manifestation of HZ and PHN is currently in late-stage development; another HZ vaccine has recently been licensed in the US and in Europe [15,16]. The licensed vaccine has demonstrated its efficacy in preventing HZ and PHN in a randomized double-blind, placebo-controlled trail involving almost 40,000 individuals 60 years of age and older. HZ and PHN-incidences were reduced in this study by approximately 51% and 67%, respectively [17]. As of yet the duration of protection was demonstrated to last at least 7 years [18]. Furthermore, the vaccine has demonstrated an acceptable safety profile in individuals 50 years and above and was as immunogenic as in people 60 years and older [19]. Based on these findings, the European Medicine Agency approved the first HZ vaccine in 2006 for immune-competent individuals sixty years of age and older, which was later changed to 50 years of age and older [16].

Germany does not have a national surveillance system in place for HZ, and therefore country-level data on the epidemiology of HZ and PHN are scarce. To support the decision making process related to a potential HZ-vaccination recommendation in the elderly we aimed to estimate annual HZ and PHN disease burden in people aged ≥ 50 years in Germany by utilizing various data sources. Since routine childhood varicella vaccination has been carried out in Germany since 2004, baseline HZ disease burden data are also urgently needed to monitor the impact of varicella vaccination on HZ epidemiology [20,21].

## Methods

### Study design

We assessed HZ-incidence (number of cases per 1,000 person-years, PY) in Germany by utilizing different data bases containing routine patient-related diagnosis data. All individuals aged ≥ 50 years with a diagnosis of acute HZ in 2007 or 2008 identified in the data bases were included in the analysis and stratified by 5-year age-groups and gender. Since the immune status was not documented in any of the utilised data bases, immune-compromised individuals were not excluded from our analyses.

### HZ disease burden in outpatients

The Association of Statutory Health Insurance Physicians (ASHIP) database contains nationwide routine outpatient data, documented by each of the 17 regional ASHIPs in Germany. The ASHIP covers all patients who are insured in the statutory health insurance (SHI) in Germany. In the SHI approximately 90% of all people living in Germany are insured. All practitioners send billing data for medical services to their regional ASHIP on a quarterly basis. These data were pseudonymized and imported into a SQL server database at the Robert Koch Institute (RKI). It contains the patients' age, gender, year and quarter of diagnosis, International Classification of Diseases (ICD)-10 codes, reliability of diagnosis as indicated by the physician (assured or suspected diagnosis, status after disease, disease excluded, or unknown), and ASHIP region. All patients with a HZ-related diagnosis code (ICD-10: B02.*) and with an 'assured' or 'suspected' reliability of diagnosis were included in the analysis (Table 1). Individuals with an undocumented gender were distributed proportionally to both genders according to the gender-proportion among individuals with available information in each age-group.

In our analysis we utilized a sample that covered in 2007 approximately 69% (14 ASHIPs) and in 2008 approximately 52% (eleven ASHIPs) of the total German population, respectively. For each of the two years we assessed the incidence of acute HZ treated as outpatients by using a cohort design: Since patients have a unique identifier in the ASHIP dataset they can be individually followed up over time. Therefore, to identify and exclude non-acute HZ-cases in 2007 we used the ASHIP dataset of 2006, and to identify non-acute outpatient HZ-cases in 2008 we used the 2007-dataset. Results from both years were extrapolated from the samples to the total population in Germany. Afterwards a weighted average was calculated for the incidence and number of HZ cases on a national level for the study period 2007-2008.

### HZ-associated hospitalization and mortality

We used the information system of the Federal Health Monitoring System (FHM) to assess the annual number of HZ-associated deaths and HZ-inpatients and used these data to calculate the mortality and incidence of HZ leading to hospitalisation in people aged ≥ 50 years. The FHM contains more than 605 million data from over 100 sources provided by the statistical offices of the federal states and the federation as well as data from the federal ministry of health, statutory health insurances, and the Robert Koch Institute [22,23].

The federal statistic office supplies ICD-10 related main diagnosis hospital statistics and ICD-10 related statistics on the primary cause of death in Germany. The hospital statistics relate to a broad base including data of all inpatients in all German hospitals. Among others, characteristics like gender, month and year of birth, date of hospitalization and discharge, and main diagnosis (ICD-10 code) are available for each patient [23]. For each death reported in the FHM system, information on gender, age group, and the ICD code of the primary disease of the deceased person (as documented by the physician who recorded the death) are documented [23].

We extracted number of hospitalized patients with a main diagnosis of HZ and the number of deaths, where the primary disease of the deceased person was HZ, in persons ≥ 50 years of age for 2007 and 2008, stratified by age and gender, and calculated a weighted average for our study period 2007-2008.

### HZ-associated complications and quality of life loss

Since there was a lack of patient-related information on PHN in the available data sources, we estimated the total number of PHN patients in Germany by applying a PHN-proportion (i.e. the average proportion of HZ-patients developing PHN) to the estimated total number of HZ-outpatients in 2007-2008. For each age-group (50-54, 55-59, etc.) we used a minimum and maximum estimate of the PHN-proportion based on available literature. Since there is no unique definition of PHN, we defined PHN as pain lasting at least three months after HZ rash onset (PHN_3_), which is commonly used in literature [17,24-30]. We identified three publications indicating the proportion of HZ-cases developing PHN_3_: Opstelten et al. found a proportion of 0.8% in 50-54 year-olds, which increased to 9% in HZ-patients aged ≥ 90 year [27]. Schiffner-Rohe et al. presented a proportion of 4.4% among 50-54 year-old HZ-patients which increased to 7.7% in patients 90 years and older [28]. Finally, Gauthier et al. found a range in HZ-patients from 8% (50-54 year-olds) to 19% (90 years and older) [25]. Based on these figures we calculated an upper and lower limit estimate of PHN disease burden in Germany. Other HZ-associated complications (e.g. HZ meningitis or ocular disease) were identified by the respective ICD-10 codes in the ASHIP database (Table 1).

For Germany, HZ-related quality of life (QoL) data are missing. To calculate the loss of quality-adjusted life years (QALY) caused by HZ in Germany, we multiplied the QALY loss per HZ-case found in studies from Canada, the United Kingdom, and Wales with the number of HZ-outpatients, which we identified in our ASHIP database and extrapolated to the total population in Germany [24,29,31]. Edmunds et al. found in the United Kingdom and Wales a QALY loss per HZ case of 0.01 for all age-groups 50 years and above [31]. In the other two studies, QALYs lost per HZ case increased by age from 0.01 to 0.11 and from 0.026 to 0.200 in the age-groups 50-54 to ≥ 90 years, respectively [24,29]. Based on these values, an upper and lower limit estimate for QALY loss per age-group due to HZ was calculated for the population in Germany.

### Statistical methods

We used PASW 17 (SPSS, Somers, NY), Stata 11 (Stata, College Station, TX), Microsoft Excel 2003 and Microsoft SQL Server Management Studio 2008 (Microsoft, Redmond, WA) for data management and analysis, and calculated 95% Poisson confidence intervals for all values except QALYs.

## Results

### HZ in outpatients in the study sample and country-level estimates

A total of 210,310 and 164,335 outpatients aged ≥ 50 years with an acute HZ diagnosis were identified in the ASHIP sample in 2007 and 2008 respectively (Table 2). Over all age-groups the proportion of HZ-cases with a diagnose reliability "confirmed" was about 90% (with the remaining 10% being "suspected"). Among identified HZ-cases, the proportion of individuals with an undocumented gender remained in both years below 6%. The most frequently coded ICD-10 with approximately 77% was B02.9 (HZ without complication) followed by B02.2 (HZ with other nervous system involvement) with almost 13%. HZ with ocular involvement (B02.3) was coded in approximately 4% of HZ-cases (Table 1).

**Table 1:** Number of cases and proportion of HZ diagnoses (ICD-10 codes^a^) in the weighted study sample, 2007/2008

ICD-10 Code	Number of cases^b^	Proportion (%)	Description
**B02.-**	6	**0.00**	Zoster [herpes zoster] Includes: shingles/zona
**B02.0**	734	**0.39**	Zoster encephalitis/Zoster meningoencephalitis
**B02.1**	47	**0.02**	Zoster meningitis
**B02.2**	25,654	**13.48**	Zoster with other nervous system involvement
**B02.3**	8,181	**4.30**	Zoster ocular disease
**B02.7**	1,100	**0.58**	Zoster generalisatus
**B02.8**	7,122	**3.74**	Zoster with other complications
**B02.9**	147,514	**77.49**	Zoster without complication (e.g. Zoster NOS)

**total**	190,358	**100.00**	

NOS = not otherwise specified

^a^World Health Organisation [46]

^b^Weighted average from study samples 2007 and 2008 - without extrapolation

Extrapolating results of the study sample to the total German population aged ≥ 50 years revealed 300,511 cases and 312,002 HZ outpatient cases for 2007 and 2008, respectively. These figures result in a weighted annual average of 306,511 patients or 9.60 per 1,000 PY treated for acute HZ in an outpatient facility in Germany 2007-2008 (Table 3). HZ-incidence increased with age: In the age-group 50-54 years average HZ outpatients incidence was 6.21 per 1,000 PY and increased to 13.19 in persons 90 years and older. Comparing individuals in their 70s with people between 50 and 54 years of age the incidence almost doubled within two decades (Table 3).

**Table 2:** Number of Herpes Zoster (HZ) outpatient cases and HZ-incidence in the study sample, 2007 and 2008

	Outpatient HZ cases (study sample) 2007				Outpatient HZ cases (study sample) 2008			
**Age-group (years)**	**Population**	**Cases**	**Incidence^a^**	**CI (95%)**	**Population**	**Cases**	**Incidence^a^**	**CI (95%)**

**50 - 54**	3,751,003	23,285	**6.21**	6.13 - 6.29	2,881,754	18,396	**6.38**	6.29 - 6.48
**55 - 59**	3,435,593	26,224	**7.63**	7.54 - 7.73	2,671,483	20,655	**7.73**	7.63 - 7.84
**60 - 64**	2,799,252	25,341	**9.05**	8.94 - 9.17	2,136,220	19,224	**9.00**	8.87 - 9.13
**65 - 69**	3,673,692	39,190	**10.67**	10.56 - 10.77	2,688,120	29,172	**10.85**	10.73 - 10.98
**70 - 74**	3,010,534	33,776	**11.22**	11.10 - 11.34	2,430,392	27,943	**11.50**	11.36 - 11.63
**75 - 79**	2,229,980	26,820	**12.03**	11.88 - 12.17	1,644,341	20,252	**12.32**	12.15 - 12.49
**80 - 84**	1,598,890	19,912	**12.45**	12.28 - 12.63	1,233,658	15,603	**12.65**	12.45 - 12.85
**85 - 89**	889,002	10,935	**12.30**	12.07 - 12.53	731,191	9,375	**12.82**	12.56 - 13.08
**90+**	376,927	4,827	**12.81**	12.45 - 13.17	269,752	3,715	**13.77**	13.33 - 14.22
**total**	**21,764,873**	**210,310**	**9.66**	**9.62 - 9.70**	**16,686,911**	**164,335**	**9.85**	**9.80 - 9.90**

CI = confidence interval

^a^cases per 1,000 person-years

Females were significantly more frequently affected by HZ. Of all HZ-cases identified in the ASHIP samples approximately 65% were females. Overall HZ-incidence in females and males was estimated at 11.12 and 7.80 per 1,000 PY, respectively. The age-dependency of the incidence existed in both genders (Table 3).

**Table 3:** Weighted annual average HZ-incidence extrapolated on the total German population^a^, 2007/2008

	Total				Male		Female	
**age group (years)**	**Population**	**Cases**	**Incidence^b^**	**CI (95%)**	**Incidence^c^**	**CI (95%)**	**Incidence^d^**	**CI (95%)**

**50 - 54**	5,831,377	36,220	**6.21**	6.15 - 6.28	**4.64**	4.57 - 4.72	**7.80**	7.70 - 7.90
**55 - 59**	5,305,382	40,274	**7.59**	7.52 - 7.67	**5.84**	5.75 - 5.94	**9.31**	9.19 - 9.43
**60 - 64**	4,225,970	37,769	**8.94**	8.85 - 9.03	**7.22**	7.11 - 7.34	**10.59**	10.46 - 10.73
**65 - 69**	5,218,814	55,816	**10.70**	10.61 - 10.78	**8.95**	8.84 - 9.07	**12.30**	12.17 - 12.43
**70 - 74**	4,362,971	49,473	**11.34**	11.24 - 11.44	**9.87**	9.74 - 10.01	**12.59**	12.44 - 12.73
**75 - 79**	3,014,860	36,633	**12.15**	12.03 - 12.28	**10.96**	10.78 - 11.15	**13.02**	12.85 - 13.19
**80 - 84**	2,219,495	27,810	**12.53**	12.38 - 12.68	**11.35**	11.11 - 11.59	**13.15**	12.96 - 13.33
**85 - 89**	1,247,016	15,684	**12.58**	12.38 - 12.78	**11.65**	11.29 - 12.03	**12.91**	12.68 - 13.15
**90+**	518,045	6,832	**13.19**	12.88 - 13.51	**11.99**	11.39 - 12.61	**13.58**	13.21 - 13.96

**total**	**31,943,930**	**306,511**	**9.60**	**9.56 - 9.63**	**7.80**	**7.75 - 7.84**	**11.12**	**11.07 - 11.17**

CI = confidence interval

^a^weighted average based on extrapolated study samples 2007 and 2008

^b^cases per 1,000 person-years

^c^cases per 1,000 males and year

^d^cases per 1,000 females and year

### Hospitalization and mortality due to HZ

The FHM data base contained 14,181 and 14,273 inpatient HZ-cases in 2007 and 2008, respectively. This results in a weighted average of 14,249 HZ-related inpatients in the study period 2007-2008. Between age-group 50-54 and 90+ years, incidence of HZ-associated hospitalization increased from 0.13 to 1.08 per 1,000 PY (Table 4). Of hospitalized HZ-cases aged ≥ 50 years 62% were female. The incidence of HZ leading to hospitalisation was 0.51 per 1,000 PY in females and 0.38 in males. An age-dependent increase in hospitalization incidence was identified in both males and females (Table 4).

**Table 4:** Weighted annual average of total number of cases and incidence of Herpes Zoster (HZ) leading to hospitalization, Germany, 2007/2008

	Total				Male		Female	
**age group (years)**	**Population**	**Cases**	**Incidence^a^**	**CI (95%)**	**Incidence^b^**	**CI (95%)**	**Incidence^c^**	**CI (95%)**

**50 - 54**	5,831,377	754	**0.13**	0.12 - 0.14	**0.11**	0.10 - 0.18	**0.14**	0.13 - 0.16
**55 - 59**	5,305,382	1,040	**0.20**	0.18 - 0.21	**0.16**	0.15 - 0.18	**0.23**	0.21 - 0.25
**60 - 64**	4,225,970	1,296	**0.31**	0.29 - 0.32	**0.28**	0.25 - 0.30	**0.34**	0.31 - 0.36
**65 - 69**	5,218,814	2,299	**0.44**	0.42 - 0.46	**0.41**	0.38 - 0.43	**0.47**	0.45 - 0.50
**70 - 74**	4,362,971	2,394	**0.55**	0.53 - 0.57	**0.52**	0.49 - 0.56	**0.57**	0.54 - 0.60
**75 - 79**	3,014,860	2,381	**0.79**	0.76 - 0.82	**0.76**	0.71 - 0.81	**0.81**	0.77 - 0.86
**80 - 84**	2,219,495	2,196	**0.99**	0.95 - 1.03	**0.92**	0.85 - 0.99	**1.03**	0.98 - 1.08
**85 - 89**	1,247,016	1,330	**1.07**	1.01 - 1.13	**1.00**	0.89 - 1.11	**1.09**	1.03 - 1.16
**90+**	518,045	559	**1.08**	0.99 - 1.17	**0.90**	0.74 - 1.08	**1.14**	1.04 - 1.25

**Total**	**31,943,930**	**14,249**	**0.45**	**0.44 - 0.45**	**0.38**	**0.37 - 0.39**	**0.51**	**0.50 - 0.52**

CI = confidence interval

^a^cases per 1,000 person-years

^b^cases per 1,000 males and year

^c^cases per 1,000 females and year

For 2007 and 2008, in total 69 and 56 deaths were reported to be caused by HZ, respectively. The weighted average was 66 individuals aged ≥ 50 years, who were reported as deaths due to HZ in 2007-2008, which translated in a mortality of 0.21 per 100,000 PY. Forty-eight (73%) of the deceased HZ-patients were ≥ 80 years of age, and mortality increased with age from 0.02/100,000 (age-group 50-54) to 3.86/100,000 (age-group ≥ 90 years) (Table 5). The number of deaths was more than 3-times higher in females, with a weighted average of 51 compared to 15 in males.

**Table 5:** Annual deaths, PHN_3_^a^ incidence and QALY^b^ loss due to Herpes Zoster (HZ) in Germany^c^, 2007/2008

	Deaths			PHN incidence based on different szenarios^d^				QALY loss due to HZ	
				**Min**		**Max**			
**age group (years)**	**Deaths**	**mortality rate^e^**	**CI (95%)**	**Incidence^f^**	**CI (95%)**	**Incidence^f^**	**CI (95%)**	**Min^g^**	**Max^g^**

**50 - 54**	1	**0.02**	0.00 - 0.10	**0.05**	0.04 - 0.06	**0.50**	0.48 - 0.52	362	942
**55 - 59**	0	**0.00**	NA	**0.22**	0.21 - 0.23	**0.76**	0.74 - 0.78	403	1,047
**60 - 64**	1	**0.02**	0.00 - 0.13	**0.26**	0.24 - 0.28	**0.98**	0.95 - 1.01	378	2,531
**65 - 69**	3	**0.06**	0.01 - 0.17	**0.35**	0.34 - 0.37	**1.39**	1.36 - 1.42	558	3,740
**70 - 74**	5	**0.11**	0.04 - 0.27	**0.37**	0.36 - 0.39	**1.70**	1.66 - 1.74	495	5,442
**75 - 79**	9	**0.30**	0.14 - 0.57	**0.78**	0.75 - 0.81	**2.19**	2.14 - 2.24	366	4,030
**80 - 84**	12	**0.54**	0.28 - 0.94	**0.96**	0.92 - 1.01	**2.63**	2.56 - 2.70	278	5,562
**85 - 89**	15	**1.20**	0.67 - 1.98	**0.97**	0.92- 1.03	**2.39**	2.31 - 2.48	157	3,137
**90+**	20	**3.86**	2.36 - 5.96	**1.02**	0.93 - 1.11	**2.51**	2.37 - 2.65	68	1,366

**total**	**66**	**0.21**	**0.16 - 0.26**	**0.43**	**0.43 - 0.44**	**1.33**	**1.32 - 1.34**	**3,065**	**24,094**

CI = confidence interval; NA = not applicable

^a^postherpetic neuralgia lasting over three months after HZ diagnosis.

^b^quality-adjusted life year.

^c^extrapolation on living population in Germany based on study sample

^d^data based on Schiffner-Rohe et al. 2010 [28], Gauthier et al. 2009 [25], Opstelten et al. 2002 [27].

^e^deaths per 100,000 person-years.

^f^cases per 1,000 person-years.

^g^data based on van Hoek et al. 2009 [29], Brisson et al. 2008 [24], Edmunds et al. 2001 [31].

### Complication and quality of life loss

When applying upper and lower limit estimates for the proportion of HZ-cases developing PHN by age-group, the overall PHN-incidence for Germany was estimated to range between 0.43 and 1.33 per 1,000 PY in individuals aged ≥ 50 years (Table 5). The annual QALY loss due to HZ in individuals 50 years and older in Germany was estimated to range between 3,065 and 24,094 (Table 5).

## Discussion

Our study provides the first nationwide estimates of annual disease burden caused by HZ and PHN in Germany. We derived these estimates from a comprehensive physician database that covers approximately 60% of the total German population 50 years of age and older. With an annual incidence of almost 1% in persons aged ≥ 50 years, HZ poses a considerable burden to the health care system in Germany both in terms of outpatient and inpatient services. The increase in disease incidence and HZ-associated mortality with age reconfirms the remarkable affection of the elderly. And due to the demographic aging in Germany it is likely that HZ disease burden will further increase in the future.

The magnitude of incidence observed in our study is consistent with previously published reports from Germany and other European countries. In 1992/93, HZ-incidence in the district of Ansbach, Germany, was found to be 3.41 per 1,000 PY in people ≥ 50 years, and a study conducted in the German federal state of Hesse revealed an incidence of 9.40 per 1,000 PY in 2004 [28,32]. Studies from other European countries demonstrated incidences ranging from 5.23 to 8.93 per 1,000 in the same age-group [25,26,31,33,34]. In Australia, an incidence range of 9,70 to 10.10 HZ-cases per 1,000 PY in people 50 years and over was reported [35]. A randomised clinical trial, conducted 1998-2001 in the US to assess the efficacy of a HZ candidate vaccine, revealed an incidence of 11.12 per 1,000 PY but including only individuals aged ≥ 60 years [17]. A systematic literature review concerning HZ incidence in people 60 years and over identified an incidence range of 3.6 to 14.2 per 1,000 PY [36]. Even though the incidences observed in different study settings were in a rather similar magnitude, the burden posed to the healthcare systems in the individual countries might vary considerably due to difference in the systems.

The remarkable increase in HZ-incidence by age can be explained with waning VZV-immunity [2,3]. The observed difference in sex-distribution remains, however, unclear. Several other studies reported in accordance to our assessment a higher incidence in females, e.g. in France, Germany, Italy, and the US [17,25,26,28,34,37,38]. It has been suggested, that there might be a difference in the immune response to latent viral infection among women and men, but the exact mechanisms are unknown [38].

An average annual 66 deaths due to HZ was reported within the FHM database we utilized, resulting in a mortality of 0.02 deaths per 100,000 PY (age-group 50-54) to 3.86 deaths per 100,000 PY (in 90 years and over), which is lower than estimated in France, where mortality ranges from 0.072 (age-group 55-64 years) to 19.48 deaths per 100,000 PY in people over the age of 94 [26]. Differences in the reporting system might be a reason for this discrepancy.

Our assessment of QALYs is limited by the fact that HZ-related QoL estimates for Germany were not available. However, the countries from where the QoL estimates were derived seem fairly similar to Germany as far as demographics and health care standard are concerned [24,29,31]. Values concerning QALY loss may differ intensely as they depend on epidemiology, population, evaluation technique, and the general setting in which those values were surveyed [39]. We accounted for the resulting uncertainty by calculating a range consisting of lower and upper limit estimates. Annual QALY losses caused by other major diseases in Germany have been published previously. For example, osteoporosis-attributable hip fractures were estimated to cause more than 66,000 QALYs lost in Germany in 2002, and obesity almost 370,000 QALYs [40,41]. Due to the fact that high disease incidence leading to considerable QALY loss, HZ can be regarded - even without considering QALY loss caused by PHN - as a relevant public health problem in Germany.

The use of the ASHIP and FHM databases may, however, cause some limitations. None of the two databases provided information on PHN, which is the most important complication of HZ and therefore crucial for estimating HZ disease burden [6,31]. By utilizing published literature we compensated this data gap. The true PHN_3_-incidence for the population in Germany is likely to be within the range identified by using the results of the published studies. Since ASHIP-data were also available for 2006 and patients can be tracked by unique identifier, we were able to identify and include only acute HZ cases in 2007 to prevent overestimation of HZ-incidence. However, for 28% of HZ-cases in 2008 the classification as an acute case was not possible as we were not able to track them in 2007 due to the different regional datasets being available. This could cause a slight overestimation of HZ-incidence in 2008. Finally, the inpatient incidence might be slightly overestimated when using the FHM database, since we were not able to exclude non-acute HZ cases. An underestimation might have occurred on the other hand, since inpatients with HZ as a secondary diagnosis were not included in our analysis, but still can contribute to prolonged hospitalizations and additional costs. However, the strength of our study lies within the data sources we used. The ASHIP database provides high-quality outpatient information from a considerable proportion of the German population and can be utilized in a cohort-design fashion; the FHM-System provides data from a number of databases in the German health care system.

Germany is one of the few countries worldwide where routine childhood varicella vaccination is recommended, with its initiation in 2004 and a change from a recommended one-dose to a two-dose vaccination schedule in 2009 [42]. Mathematical modelling suggests that routine childhood varicella vaccination will reduce VZV-circulation in nature, which will decrease the number of natural boosters of VZV-immunity in the population. According to mathematical models, this might lead to a substantial increase in HZ-incidence in the population for 40 to 50 years after the initiation of routine varicella vaccination [43,44]. Since mathematical models are based on many assumptions, it is important to closely observe whether this phenomenon will occur in nature. Since 2005 a physician sentinel-system is in place in Germany to monitor the effects of the current varicella vaccination recommendation [42]. As of today no clear increase in reported HZ-cases per doctors' practices has been observed. But due to the lack of a proper denominator, HZ incidences can not be determined [42]. In the United States, where a varicella vaccination program is in place since 1995, various studies have as of yet also not shown a clear effect of the program on HZ-incidence, even though the incidence of varicella declined rapidly [21,45].

To monitor potential changes in HZ-epidemiology, countries with routine varicella vaccination should assess HZ-incidence on a periodical basis. HZ incidence and disease burden data presented in this manuscript will serve as important baseline data, and subsequent follow-up incidence data will not only be of critical importance to the German health system, but also for other countries who have recently introduced or are currently considering the introduction of routine childhood varicella vaccination.

## Conclusions

Our study confirms the considerable disease burden that HZ and PHN place on the German health care system. With an aging population it is likely that HZ disease burden will continue to increase in Germany, and the implemented routine childhood varicella vaccination program might contribute to an increase in disease burden in the future as well. Our study provides important baseline estimates of HZ-incidence in different age-groups in Germany, which will be important also for the development of a HZ vaccination recommendation. Follow-up periodical analyses of ASHIP-data will be performed to monitor the impact of varicella and herpes zoster vaccination in Germany.

## Competing interests

Potential conflicts of interest: Before initiation of this research, BU was an intern at GlaxoSmithKline from October 2006 to September 2007 and an employee of Sanofi Pasteur MSD (provider of a herpes zoster vaccine) from April 2008 to May 2010. For all other authors: No competing interests. The study was conducted without financial support.

## Authors' contributions

BU participated in the study design, analysis of data, and drafted the manuscript. TRi participated in the data preparation and manuscript review. AS, TRe and GK participated in the study design and reviewed the manuscript. OW participated in study design, analysis of data, and review of the manuscript. All authors validated the design and final results of the study. All authors read and approved the final draft manuscript.

## Pre-publication history

The pre-publication history for this paper can be accessed here:

http://www.biomedcentral.com/1471-2334/11/173/prepub
